# In-memory computing on a photonic platform

**DOI:** 10.1126/sciadv.aau5759

**Published:** 2019-02-15

**Authors:** Carlos Ríos, Nathan Youngblood, Zengguang Cheng, Manuel Le Gallo, Wolfram H. P. Pernice, C. David Wright, Abu Sebastian, Harish Bhaskaran

**Affiliations:** 1 Department of Materials, University of Oxford, Parks Road, Oxford OX1 3PH, UK.; 2 IBM Research–Zurich, Säumerstrasse 4, 8803 Rüschlikon, Switzerland.; 3 Institute of Physics, University of Muenster, Heisenbergstr, 11, 48149 Muenster, Germany.; 4 Department of Engineering, University of Exeter, Exeter EX4 QF, UK.

## Abstract

Collocated data processing and storage are the norm in biological computing systems such as the mammalian brain. As our ability to create better hardware improves, new computational paradigms are being explored beyond von Neumann architectures. Integrated photonic circuits are an attractive solution for on-chip computing which can leverage the increased speed and bandwidth potential of the optical domain, and importantly, remove the need for electro-optical conversions. Here we show that we can combine integrated optics with collocated data storage and processing to enable all-photonic in-memory computations. By employing nonvolatile photonic elements based on the phase-change material, Ge_2_Sb_2_Te_5_, we achieve direct scalar and matrix-vector multiplication, featuring a novel single-shot *Write*/*Erase* and a drift-free process. The output pulse, carrying the information of the light-matter interaction, is the result of the computation. Our all-optical approach is novel, easy to fabricate and operate, and sets the stage for development of entirely photonic computers.

## Teaser

Nonvolatile multilevel phase-change memories on integrated photonic devices enable all-optical direct in-memory multiplications.

## INTRODUCTION

Integrated photonics offers attractive solutions for using light to carry out computational tasks on a chip (*1*–*6*), and phase-change materials are emerging as functional materials of choice on photonic platforms (*7*–*13*). On-chip nonvolatile memories that can be written, erased, and accessed optically are rapidly bridging a gap toward all-photonic chip-scale information processing (*13*–*15*). However, breaking the processor-memory dichotomy would vastly transform the computing landscape by allowing processing directly on the memory elements—so-called in-memory computing. Electronic implementations of such systems, able to carry out complex tasks such as scalar multiplication, bulk-bitwise operations, correlation detection, and compressed sensing recovery, are now emerging (*16*–*20*). Photonic implementations of in-memory computing on an integrated photonic chip have the potential to further transform the computing landscape, by providing, ultimately, increased speeds and bandwidths that can come from working directly in the optical domain, leveraging both inherent wavelength division multiplexing capabilities and the rapid technological advances of the Si photonics “revolution” (*21*). Although integrated photonic memories have been showcased in recent years (*11*–*13*), carrying out computational tasks in the same device that implements the memory function provides crucial challenges in terms of switching energy, speed, and single-shot programming and recovery. Here, we overcome such challenges and demonstrate the first instance of a photonic computational memory for direct scalar multiplication of two numbers. Specifically, we demonstrate the multiplication *a* × *b* = *c*, with *a*, *b*, *c* ∈ [0, 1], using a single integrated photonic phase-change memory cell. Subsequently, we extend this idea to demonstrate matrix-vector operations using multiple phase-change cells. Note that, matrix-vector multiplication operations underpin key processing operations in the area of “big data” analytics and artificial intelligence (AI) (*16*). For example, such an architecture can be used for the efficient solution of systems of linear equations (*22*) and a host of other modern-day computational areas, such as machine learning [e.g., for sparse coding (*17*) and compressed sensing (*20*)] and deep learning [e.g., for forward and backward propagation in deep neural networks (*23*, *24*)]. Our work thus represents a key milestone for optical processing in memory.

## RESULTS

**Fig. 1:**
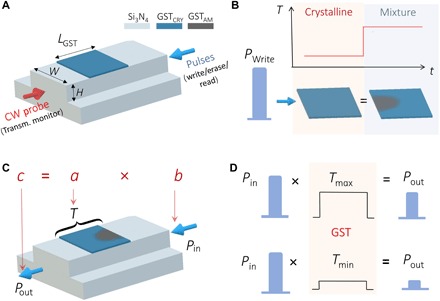
GST-based photonic memory cell and the scalar multiplication concept. (**A**) Scheme of an on-chip bidirectional pump-probe to switch, read, and monitor the transmission in a GST-based nanophotonic memory device. Write and Erase consisted of pulses at λ = 1590 nm and the probe of a low-power CW signal at λ = 1598 nm. *L*_GST_ = 1 or 2 μm, *W* = 1.3 μm, and *H* = 165 nm (etched down from a 330-nm-thick Si_3_N_4_). (**B**) The pulse *P*_Write_ is used to switch the GST from a low to a high transmission level, thus defining the transmittance *T* of the device. (**C**) Scheme of the multiplication of two scalars *a* and *b*, codified in the device transmittance *T* and in the energy of the read pulse *P*_in_. (**D**) The low-energy read pulse *P*_in_, which propagates through without inducing phase change, is measured at the output with an amplitude modulated by the transmittance *T*.

We used the photonic memory device with phase-change materials shown in Fig. 1A as the functional element to demonstrate scalar multiplication of two numbers. To do so, we mapped the numbers to the power of an input pulse *P*_in_ and the transmittance *T* of the device, which is set by a Write pulse *P*_Write_, as sketched in Fig. 1 (A to D). Our device relies on the near-field coupling between the propagating electromagnetic mode inside the waveguide and a phase-change material segment (cell) placed on top (of the waveguide) to absorb enough energy to crystallize (anneal over 150°C) or amorphize (melt-quench over ~600°C) (*25*). Because most phase-change materials have a nonnegligible imaginary refractive index in the visible and near-infrared wavelength range, light is attenuated in different amounts depending on the phase configuration of the material, which gives rise to differentiable transmitted signals, thus encoding information.

The Write pulse *P*_Write_ modulates the transmittance state of the photonic memory device. In particular, we used pulse energies $E_{P_{\text{Write}}}$ and $E_{P_{\text{in}}}$, such that $E_{P_{\text{Write}}}>E_{\text{Th}}>E_{P_{\text{in}}}$, where *E*_Th_ is the threshold energy to partially amorphize the fully crystalline phase-change material. Pulses with energies below such threshold will not change the transmittance because the temperature reached by the GST is lower than the melting point, resulting in no amorphization. Moreover, the recrystallization process requires pulses with energies in the same range as those used to amorphize; therefore, pulses with *$E_{P_{\text{in}}}<E_{\text{Th}}$* also do not have any effect on intermediate partial-amorphous states (*13*). As we demonstrate, this simple yet powerful architecture is a promising solution for optical information processing applications, given that light is attenuated without directly blocking the transmission. In our experiments, we use the phase-change material alloy Ge_2_Sb_2_Te_5_ (GST) in combination with Si_3_N_4_/SiO_2_ photonic waveguides at telecom wavelengths, as sketched in Fig. 1A (see Materials and Methods and the Supplementary Materials). To characterize our devices and condition our material for multilevel operation (i.e., the process of programming the device to specific levels), we used the counter-propagating pump-probe measurement configuration we had previously utilized (*13*). Pulses were used to control the phase configuration of GST, while a low-power continuous-wave (CW) probe was used to monitor the transmission state. Multiple and nonvolatile levels of transmission were reached, as a result of the mixture between amorphous and crystalline GST (*26*), by controlling the power of the pump pulse to Write (amorphize) up to any level of higher transmission. To Erase (recrystallize), we used two approaches: a train of decreasing-energy pulses as used in (*10*, *13*) and, as explained later in this paper, a single two-step pulse. The former allowed us to reach any of the lower transmission levels, while the latter allowed us to recrystallize down to the baseline. While using the train of pulses, there are no restrictions in intralevel transitions, i.e., any level can be reached starting from any other and the number of levels is limited by the geometry of the GST cell, the available signal-to-noise ratio (SNR), and the inherent randomness associated with phase transition in GST (*27*).

Figure 1B shows the Write pulse, *P*_Write_, used to program a specific level of transmittance *T* of the device, which relies on the multilevel conditioning of the material. The portion of the *P*_Write_ pulse that is transmitted was not taken into account (in the calculation of the result of the multiplication). A second low-energy read pulse *P*_in_ propagates through the photonic memory device, experiencing a transmittance given by the current phase configuration of the GST cell (as conditioned by the pulse *P*_Write_), but does not induce any change to the material. The power of the pulse of *P*_in_ at the output port, *P*_out_ = *T*(*P*_Write_) × *P*_in_, is the result of the multiplication *a* × *b* by mapping the multiplicand *a* to *T* and the multiplier *b* to *P*_in_, as shown schematically in Fig. 1C and in more detail in Fig. 1D. Such a direct multiplication process is considerably more efficient than previous demonstrations of multiplication by sequential addition (*15*, *28*, *29*).

**Fig. 2:**
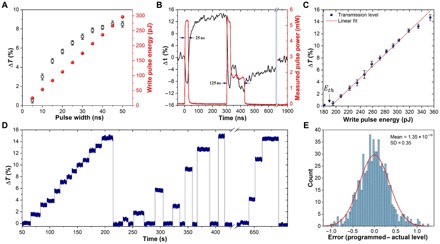
Device optimization. (**A**) Write pulses with varying widths achieve different transmission levels in a 1-μm-long GST cell before saturating at around 45 ns and their equivalent pulse energy. Shorter pulses can be chosen, thus saving energy and time during memory operations. Δ*T* = (*T* − *T*_min_)/*T*_min_ is the change in transmission of the level *T* with respect to the baseline *T*_min_. (**B**) Amorphization to a higher transmission level is achieved with a single square Write pulse (*13*), while a new Erase double-step pulse is used to fully recrystallize, i.e., bring the transmission to the baseline. The latter pulse, at the input, consisted of 14.1 mW (or peak power) for 25 ns and 5.64 mW (0.4 times the peak power) for 100 ns, where peak power is equal to the power used to reach the maximum transmission level. (**C**) Write pulse energies used for the multilevel conditioning shown in (D) with a linear response by the GST memory cell beyond the threshold energy. (**D**) Multilevel conditioning of a 2-μm-long GST cell. Thirteen distinguishable levels are demonstrated and accessed randomly; the number of levels is limited only by the SNR and the confidence interval as shown in (E). (**E**) Error of the multilevel operation calculated from subtracting the measured transmission level from the programmed level in 600 transitions or switching events. The red line corresponds to a normal distribution fitting curve.

We first optimized the parameters of our devices, particularly to decrease the energy consumption and to improve the speed of operation. For a chip with a 1-μm-long cell of GST, the pump pulse width was varied while keeping a constant power to reach different transmission levels, as shown in Fig. 2A. For pulses with widths longer than 45 ns, there is a saturation due to the finite size of our memory cell, in which no more amorphous material can be obtained without using higher powers (*30*, *31*). Therefore, longer pulses represent a waste in energy as the GST will not further amorphize and could even be ablated. From these results, it can also be observed that 25-ns pulses induce a change equivalent to ~75% of the maximum achievable transmission. We found that for cells between 1 and 4 μm long, 25-ns pulses were enough to achieve clear transmission contrast among levels, limited only by the SNR of the measurement. We further reduced the energy consumption by using single-shot Erase pulses from any arbitrary level to the fully crystalline level (baseline). This was achieved by using the double-step pulse sequence, shown in Fig. 2B, using a total energy of approximately 917 pJ, of which only ~577 pJ was absorbed by GST (also known as switching energy). The double-step pulse is sufficient to heat the material over the crystallization temperature and of a duration (125 ns) sufficient to keep it above the glass transition temperature, but below the melting temperature, until full recrystallization occurs. This double-step Erase pulse replaces the multiple-pulse erase scheme in previous works [e.g., the 19 × 100–ns pulse scheme in Ríos *et al*. (*13*)] where each individual pulse induced partial recrystallization. The double-step pulse approach results in a considerable reduction in energy consumption and increase in overall speed during the Erase process [again, for example, in Ríos *et al*. (*13*)]; the total energy used during the Erase process was 9.5 nJ, and the duration was 3.8 μs, figures that are improved upon by factors of 10 and 25, respectively, in the current work. In terms of operational speed for a Write/Erase cycle, it can be observed in Fig. 2B that approximately 200 ns (including the pulse duration) is required to obtain a stable transmission level for the Write pulse and ~600 ns for the Erase pulse. However, this time varies with the pulse width: The shorter the pulse, the lower the time for the material to cool down, and for the refractive index to stabilize, the timescale of which is governed by the thermo-optical effect (*32*). Further improvements in terms of speed and energy can be achieved by decreasing the pulse width to even shorter times, especially the Write pulse, which could be a picosecond and even femtosecond pulse (*10*, *33*, *34*). This would also reduce the “dead” time between the two pulses used to perform the scalar multiplication (see below).

Since the ability to program our integrated phase-change photonic cells into multilevel states is key to the implementation of direct scalar multiplication, we demonstrate in Fig. 2 (C and D) multilevel behavior in a 2-μm-long GST cell using single-shot Write and Erase with the same pulse parameters of Fig. 2B. Thirteen transmission levels were programmed, limited only by the noise of the photodetector (see the Supplementary Materials). We found a linear response of the attenuation caused by the GST cell as the pulse energy is increased beyond the threshold energy. For large energies, the linearity is lost due to saturation, which implies that the GST is in its most amorphous state. The beginning of such saturation can be observed in Fig. 2C for pulse energies approaching 354 pJ. For this reason, only energies of up to 354 pJ were considered here. To study the error in reaching the programmed transmission level, we sent combinations of Write and Erase pulses to reach 10 different transmission levels in 20 consecutive sequences. This experiment was repeated three times. The results of subtracting the actual transmission level after a particular transition from the originally programmed level are plotted in the Fig. 2E. The programmed levels were taken as the average level after several back-and-forth switching during a conditioning process (*13*). With an SD of 0.35% (for the total change in transmission for Δ*T*), a confidence interval for each transmission level can be established so that each level is uniquely distinguished. This value is determined not only by SNR but also by the variation in power of the pump pulses due to fluctuations in the electro-optical conversion.

**Fig. 3:**
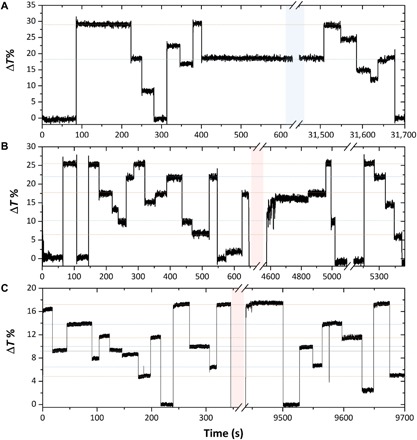
Drift in transmission. (**A**) Multilevel operation of a 2-μm-long GST memory cell using 50-ns pulses with energies in the range of 350 to 600 pJ to Write (upward transitions) and a train of power-decreasing pulses to Erase (downward transitions) level (*13*). The blue highlighted area corresponds to ~8.5 hours of constant measurement with a CW probe of 0.1 mW (inside the waveguide). (**B**) Same as (A), but turning the probe OFF for around 1.5 hours. A drift is observed between 4600 and 4800 s, which is corrected by sending the pulse energy of the level where the memory was originally set. (**C**) Multilevel operation of a different 2-μm-long GST memory cell using 25-ns pulses with energies in the range of 200 to 354 pJ (see Fig. 2D). The Write and Erase were done in the same way as for (A) and (B). The probe was turned off for a time of ~2 hours, but in this case, the CW probe power was reduced to 0.05 mW, which is enough to avoid drift. The colored traces are added to track the evolution of the levels.

For the proper execution of scalar multiplication operations using multilevel memory states, it will also be important for such states to be stable over time. Thus, we show in Fig. 3 the optical transmission traces measured for a duration of over 3 × 10^4^ s (8.8 hours), with the aim of studying the temporal variations of programmed levels (drift). Transmission levels in an arbitrary order were written and erased in two 2-μm-long GST memory cells. Subsequently, the devices were kept in an intermediate transmission level for a prolonged period of time, one having a 0.1-mW probe ON and the other OFF, as shown in Fig. 3 (A and B), respectively. Mechanical drift of the sample stage was observed because of the relaxation of the picomotors over time; however, once the chip was placed in a stable position, the transmission level remained constant for the case in which the probe was kept ON. Therefore, we do not find measurable drift for up to 10^4^ s of constant measurement. This drift-free process represents a big advantage for photonic computational memory over its electrical analog, which undergoes notable resistance drift over time, thus preventing (or at least making it very difficult) to achieve and maintain reliable levels (*35*–*38*). The electrical resistance is sensitive to the Fermi level and trap states, which, for a highly stressed amorphous GST after a quick melt quenching, are sensitive to the structural relaxation that takes place over time (*38*). There is also some experimental evidence that the refractive index and the bandgap of amorphous phase-change material are also influenced by structural relaxation (*37*, *39*). The crystalline state, on the other hand, is stable and, thus, has a small or negligible resistance drift (*35*). To date, the reason the programmed levels in our optical memory devices exhibit such excellent temporal stability is not fully understood and needs further investigation. However, the temporal stability can no doubt be partially attributed to the fact that transmission is dominated by the more stable crystalline phase [even the less absorptive state of our memory cell is composed of an amorphous area embedded within crystalline material (*31*)].

Moreover, the same specific transmission levels were retrieved after the measurement; hence, the multilevel conditioning is also preserved over time. On the other hand, for the case when the probe was turned OFF, a drift of nearly 9% was observed once the probe was turned on again. This drift is due to the relaxation of the material when the probe is removed, given that the probe itself heats up the material to a constant temperature to cause a thermo-optical effect that modifies the values of the complex refractive index without crystallizing (*32*). However, once the probe was turned ON, the drift is easily corrected for by sending a write pulse with the same energy as that required to reach the originally programmed level (i.e., the level set immediately prior to the probe being turned off). As result, we are able to reliably retrieve both the original level and any other level, demonstrating that the multilevel conditioning was preserved. We were also able to avoid the drift entirely (i.e., whether the probe remains ON or OFF) by simply decreasing the probe power to 0.05 mW—half of its previous value. In this case, the material relaxation after the probe is turned OFF and then ON is negligible, given that the temperature is lower at the GST, as shown in Fig. 3C.

**Fig. 4:**
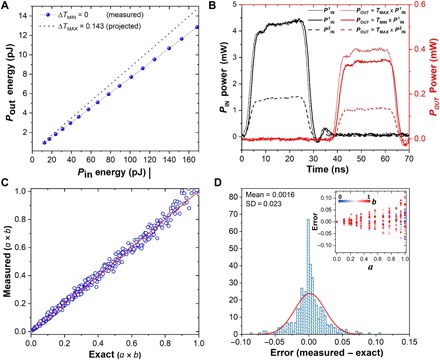
Scalar multiplication. (**A**) Read pulse *P*_in_ energies and corresponding measured *P*_out_ energy (past the crystalline GST cell and a grating coupler). All the measurements were carried out with the memory cell in the transmission baseline (i.e., Δ*T* = 0) of the multilevel conditioning shown in Fig. 2C. After each switching, an Erase pulse was used to bring the device to the baseline before the following pulse. A projection of the values for *P*_out_ for the case Δ*T* = 0.143 is plotted for comparison. (**B**) Demonstration of three multiplications by applying two different *P*_in_, *P*^1^_in_ = 112.8 pJ (~4.51 mW for 25 ns) and *P*^2^_in_ = 38.9 pJ (~1.56 mW for 25 ns), to memories programmed in the maximum and minimum transmittance levels, Δ*T* = 0.143 and Δ*T* = 0, respectively. The result when multiplying by *T* = 0 corresponds to the level-specific offset. The short time delay between pulses is due to the difference in optical path between the reference pulse and the pulse that is coupled into the photonic chip, both obtained from the pump pulse using a 90/10 beam splitter. (**C**) Realization of 429, *c* = *a* × *b* multiplications equivalent to 13 different, equally spaced, values for *T* (reached with *P*_Write_ ∈ [180 pJ, 354 pJ]), thus creating 13 values for the multiplicand *a*, and 33 different values for *P*_in_ ∈ [0 pJ, 112.8 pJ] for 33 scalars corresponding to *b* (note: *a*, *b*, *c* ∈ [0, 1]). *P*_Write_ was sent first to establish a level followed by the 33 *P*_in_ pulses before changing levels again. (**D**) Error of the scalar multiplication calculated from subtracting the measured value from the exact. The red line corresponds to a normal distribution fit whose mean and SD values are shown on the plot. The inset corresponds to the error as function of the values of *a* and *b*; this shows how for values closer to 1, the error grows linearly, which, in turn, explains the spread for values close to 1 in (C).

We now demonstrate the multiplication *a* × *b* = *c*, with *a*, *b*, *c* ∈ [0, 1]. We used a photonic memory device with a 2-μm-long GST cell and the single-shot Write and Erase scheme. We mapped the multiplicand *a* to the transmittance of the device, *T* = Δ*T* + *T*_0_, where Δ*T* corresponds to the linear response of the change in transmission as function of the *P*_Write_ pulse (shown in Fig. 2C), and *T*_0_ is the baseline transmission level (fully crystalline). Subsequently, the multiplier *b* is mapped to the energy of a second pulse *P*_in_. The result of the multiplication is calculated from the output of this latter pulse, which is equivalent to *P*_out_ = *T* × *P_in_*. We generated both *P*_Write_ and *P*_in_ in the same manner by tuning the pulse power at the electro-optical modulator (see Materials and Methods). Figure 4A shows the read pulse energies used to map the multiplier and the corresponding output pulse energy after propagating past the fully crystalline GST cell and through one grating coupler. The resulting relation shows that the response of the material (i.e., its transmittance) is linear for all the *P*_in_ energies. This result, together with the linear changes in transmittance as function of *P*_Write_, demonstrated in Fig. 2C, is highly enabling because the mapping of both scalar numbers does not rely on fitting functions, which is the case of in-memory computing with electrical PCMs following a “pseudo” Ohm’s law (*16*).

We use energies for *P*_Write_ in the [180 pJ, 354 pJ] range or equivalently, Δ*T* ∈ [0, 0.143] (see Fig. 2C), and energies for *P*_in_ in the [0 pJ, 112.8 pJ] range. In Fig. 4B, we show the experimental realization of three multiplications and the actual output pulses, *P*_out_. In particular, we used the minimum and the maximum of the multiplicand: Δ*T*_min_ = 0 (i.e., *a* = 0) and Δ*T*_max_ = 0.143(i.e., *a* = 1), and the maximum ($P_{\text{in}}^{1}$) and an intermediate ($P_{\text{in}}^{2}$) energy values for the multiplier, 112.8 pJ (i.e., *b* = 1) and 38.9 pJ (*b* = 0.4), respectively. In this figure, it can be observed that the output powers have distinct energies, which can be measured and then rescaled to obtain the result of the operations: 1 × 0 = 0, 1 × 1 = 1, and 1 × 0.4 = 0.4. Note that the lowest transmission level of the three, which in theory corresponds to 0.4, is actually smaller than the level for 0. This is due to a combination of two reasons: (i) to calculate the former, we used the relatively low power pulse $P_{\text{in}}^{2}$, whereas for the latter we use the higher power pulse $P_{\text{in}}^{1}$, and (ii) even in the fully crystalline state, GST will not absorb all the light propagating down the waveguide [unless a much longer GST cell is used to substantially increase the optical attenuation (*12*)]. Therefore, there is an offset given by the transmission baseline *T*_0_, which has to be subtracted from every multiplication to enable exact linear rescaling to the [0, 1] results (see the Supplementary Materials). In Fig. 4C, we demonstrate 429 multiplications choosing arbitrary values for *a* and *b* with the associated error (difference between the exact and the measured value of *c*) shown in Fig. 4D. We found good agreement between the exact and the measured value of the multiplication, having an error that spreads as *a* and *b* get larger, as shown in the inset of Fig. 4D (see the Supplementary Materials). The exact value was calculated from the linear fits in the characterization of the device and the subsequent mapping to [0, 1] for both multiplicand and multiplier. The measured value corresponds to the average of the output pulse energy, correcting the offset and normalizing to [0, 1]. While the results of the multiplication are not exact due to factors such as the fluctuations in the values of *T*, as shown above in Fig. 2E, this kind of multiplication operation has proved useful in application areas such as machine learning (*17*). Moreover, in application domains where arbitrarily high accuracy is required, ideas such as mixed-precision computing can be used where the low precision multiply unit is used in conjunction with a high precision unit (*16*).

**Fig. 5:**
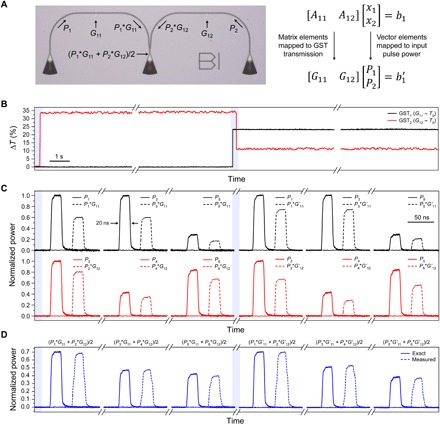
Photonic architecture for matrix-vector multiplication. (**A**) An optical image of the device used to implement multiplication between a 1 × 2 matrix and a 2 × 1 vector is shown on the left. The matrix elements are preprogrammed in the GST (*G*_11_ and *G*_12_) using two write pulses as discussed in the main text. To implement multiple vectors, pairs of *P*_in_ pulses (*P*_1_and *P*_2_, *P*_3_ and *P*_4_, etc.) are sent simultaneously and recorded as *P*_out_ (*P*_1_*G*_11_ + *P*_2_*G*_12_, *P*_3_*G*_11_ + *P*_4_*G*_12_, etc.). (**B**) Change in transmission of the two GST cells as recorded by the probe. Highlighted blue region indicates when *P*_Write_ pulses were used to switch one or more GST cells, thus reprogramming the matrix. (**C**) Recorded traces of input pulses before and after the GST cells. Transmitted power of the optical pulses (dashed lines) is the product of the input pulse and GST’s transmission. (**D**) Calculated and measured output pulses after combining pulses from the two arms on a photodetector. The different wavelengths in the two arms (1590 and 1598 nm) and linear response of the photodetector ensure that the two multiplied pulses are added together. A factor of ½ is due to the 50% loss at the waveguide splitter.

In our memory cell, the nonvolatility of the transmission levels implies that the multiplicand is fixed until the next pulse excitation changes its state. This property, in an architecture comprising arrays of scalar multiplying units, could be exploited for efficient calculation of matrix-vector multiplications. To demonstrate that this type of matrix-vector multiplication is well suited to our photonic framework, we propose an architecture to implement matrix-vector multiplication using two memory cells in parallel. In such an architecture, shown in Fig. 5A, the output signals containing the result of each multiplication—carried out in two different GST cells—are combined using a beam splitter. Initially, the transmittance values equivalent to two numbers, *G*_11_ and *G_12_*, are preprogrammed using two write pulses of power *$P_{\text{Write}}^{1}$* and *$P_{\text{Write}}^{2}$*. Given that these values are fixed, the two numbers together represent a 1 × 2 matrix [*G*_11_ *G*_12_], which can be switched to any other level at a given time, as shown in Fig. 5B. To multiply this matrix by a 2 × 1 vector, pairs of input pulses representing any two numbers, *P*_1_ and *P*_2_ for instance, are sent simultaneously to the left and right grating couplers of the device shown in Fig. 5A. After propagating through the two respective GST memory cells, the pulses are weighted by the transmittance function and, therefore, carry the information of each individual multiplication (i.e., *P*_1_ × *G*_11_ and *P*_2_ × *G*_12_). Figure 5C shows the experimental demonstration for three pairs of numbers (input pulses), which all together represent three multiplications between a constant matrix and a vector with varying elements, before and after the multiplication by the GST transmittance. Each pair of pulses is then combined using a beam splitter and summed via a photodetector with a linear response. This signal, *P*_out_, is the linear combination of the transmitted *P*_1_ and *P*_2_, provided they are optical pulses with unique wavelengths to prevent unwanted interference. The resulting scalar number is the matrix-vector product, whose outputs are shown in Fig. 5D for two different matrices (i.e., [*G*_11_ *G*_12_] and [*G*′_11_ *G*′_12_]). Good agreement is observed between the measured and the expected values, which are calculated from the GST transmittance and the measured input pulse powers, with results normalized for simplicity. This process can be generalized to a multiplication between a *k* × *N* matrix and *N* × *1* vector using multiple cascaded power splitters (see fig. S6). Using such methods, an input matrix **A** could be mapped to GST nanophotonic memory cells, and the solution ***x*** of ***Ax* = *b*** with $A\in R^{N\times N}$ and $b,x\in R^{N}$ can be computed from iterative algorithms, as demonstrated for electrically switchable phase-change memory devices (*16*). Moreover, this approach can be extended to a matrix-matrix multiplication by sequentially sending the column values of the second matrix as input vectors. The implementation of this approach can already be visualized from the results of Fig. 5, in which combining the results of the three input vectors, the following matrix-matrix multiplication can be reconstructed

$$[\begin{array}{cc} G_{11} & G_{12} \end{array}]\;[\begin{array}{ccc} P_{1} & P_{3} & P_{5}\\ P_{2} & P_{4} & P_{6} \end{array}]=\frac{1}{2}{\left[\begin{array}{c} P_{1}G_{11}+P_{2}G_{12}\\ P_{3}G_{11}+P_{4}G_{12}\\ P_{5}G_{11}+P_{6}G_{12} \end{array}\right]}^{T}$$

where the factor of ½ is due to the 50% loss at the waveguide splitter. This approach will be a time-consuming serial process if the same wavelengths for *P*_1_and *P*_2_, *P*_3_ and *P*_4_, etc., are used. A distinct advantage of photonics over the electrical domain is the ability to wavelength multiplex on-chip, so it is possible to send matrix elements *P*_1_ through *P*_6_ simultaneously and separate the respective output vector elements via wavelength filtering before summing on multiple photodetectors.

## DISCUSSION

Using the new mechanism introduced in this paper, we have shown that single sub-nanojoule energy pulses are sufficient for both Write and Erase operations in our integrated phase-change photonic devices. However, the energy consumption associated with programming matrix elements into the phase-change cells remains relatively high. It should be noted that in applications where the same matrix elements are repeatedly used, the programming step needs to be done only once, given the nonvolatile nature of information storage in the photonic memory device. In several applications such as iterative solution of linear equations, compressed sensing, and deep learning inference, the matrix elements remain fixed. The entries of the vector that is used to multiply with the fixed matrix, on the other hand, are codified into the energy of the read pulses. Hence, read pulses of energies on the order of femtojoule (pulse width of tens of picoseconds and powers of tens-hundreds of microwatts, for instance) can be used as long as the resulting output pulse can be resolved by the detector. Moreover, the collocation of processing and storage means that there is little energy expended on data transfer. Hence, the energy consumption associated with the matrix-vector multiply operation can be substantially low.

The energy consumption associated with our approach is comparable to its electronic computational memory analogs (*16*), but there are several key advantages associated with photonic in-memory computing. The most notable one is the ability to process transmitted signals optically without having to use highly inefficient electro-optical converters. The experimental results presented in this paper also point to other important advantages as follows:

(i) The output pulse varies linearly with the input (read) pulse, unlike in electrical phase-change devices (where nonlinear relationships between resistance level and excitation voltage and between voltage and current exist). This eliminates the need for nonlinear fitting functions and additional postprocessing [see, e.g., Le Gallo *et al*. (*16*)] that are needed when implementing multiplication electronically.

(ii) There is no drift of any programmed transmission level in our approach (within the test parameters presented in this paper, including continuous measurements over periods extending to 8.5 hours). This compares favorably to electrical phase-change devices, where resistance drift necessitates the use of additional processing and hardware overheads (e.g., periodic refreshing of values or the use of calibration cells).

(iii) We are able to set levels deterministically, a process that is challenging and has been the subject of much effort in electronic phase-change memories [which use iterative level-setting schemes—see, e.g., Papandreou *et al*. (*40*)].

To summarize, we have demonstrated direct multiplication of scalar numbers using photonic in-memory computing. This is achieved by using the distinct interaction of two pulses, each of which represents a number to be multiplied—one with energy above the switching threshold to induce structural changes in the phase-change material, and another one below—in an integrated photonic waveguide. We also extended this concept to matrix-vector multiplication operations that form a key computational primitive for the vast majority of AI algorithms. These results confirm the potential of phase-change materials in photonic hardware computational paradigms—including the ability to perform computations with memory in the same physical location using light. While there is plenty of room for improvement, these results are at the vanguard of collocated memory and processing on a photonic platform.

## MATERIALS AND METHODS

### Sample fabrication

The nanophotonic memory cells were fabricated on 330-nm Si_3_N_4_/3.3-μm SiO_2_ wafers. A JEOL JBX-5500ZD 50-kV electron-beam lithography (EBL) was used to write the photonic circuitry using MaN-2403 negative resist, followed by a reflow process of 90 s at 100°C. Subsequently, reactive ion etching in CHF_3_/Ar/O_2_ was carried out to etch 165 nm of the Si_3_N_4_ and thus obtain the bare photonic device_._ A second EBL writing step using poly(methyl methacrylate), followed by a lift-off process, was used to pattern the phase-change materials. A stack of 10 nm of GST with a 10-nm indium tin oxide capping (to avoid oxidation) was deposited in an argon environment using a homebuilt radio frequency sputtering system (Nordiko). Before the measurements, the GST was crystallized on a hot plate following a 5-min annealing at 250°C. Figure S1 shows an optical microscope image of the balanced splitters, the photonic device used in this work.

### Measurement setup

The pump-probe experimental setup in fig. S2 was used to carry out the multilevel, time-resolved, and scalar multiplication measurements. The setup was then extended to that in fig. S3 to carry out the matrix-vector multiplications within balanced splitters with two GST memory cells. In both setups, the optical signals were confined to the photonic circuit, that is, Write, Erase, read-out, and multiplications were all realized within the integrated chip. To avoid interference, two different C + L CW tunable laser sources were used; wavelengths of 1598 nm (TSL-550, Santec) and 1590 nm (N7711A, Keysight) were chosen for the probe and pump, respectively. The pump pulses—as well as the multiplicand pulse *P*_in_—were subsequently generated with an electro-optical modulation (Lucent Technologies, 2623NA), which was controlled by a 100-MHz electrical pulse generator (AFG 3102C, Tektronix). The pulse was further power amplified by a low-noise erbium-doped fiber amplifier (AEDFA-CL-23, Amonics). Both the pump pulses and the probe were coupled into the photonic device using integrated grating couplers with transmission peak at 1598 nm and coupling efficiency of ~20%. The counter-propagating scheme was used to ease the separation of the signals, and tunable optical filters (OTF-320, Santec) were introduced to the optical lines to further suppress noise resulting from reflections. At the probe output of the device, the CW signal was divided into two beams using a 90/10 beam splitter to measure the time-resolved and the long-term transmission with a 200-kHz low-noise photoreceiver (NewFocus, 2011) and a 125 MHz photodetector (NewFocus, 1811), respectively. At the other output, the transmitted pulses were monitored using a 1-GHz photodetector (NewFocus, 1611).

## Supplementary Material

http://advances.sciencemag.org/cgi/content/full/5/2/eaau5759/DC1

## SUPPLEMENTARY MATERIALS

Supplementary material for this article is available at http://advances.sciencemag.org/cgi/content/full/5/2/eaau5759/DC1 Section S1. Device characterizations using balanced splitters Section S2. Experimental setup Section S3. Noise Section S4. Offset correction Section S5. Error propagation Section S6. Proposed matrix-vector multiplication architecture Fig. S1. Balanced splitter characterization. Fig. S2. Diagram of the experimental pump-probe setup. Fig. S3. Diagram of the experimental setup for matrix-vector multiplication. Fig. S4. Noise for up to 20-μs measurements using a 125-MHz (New Focus, 1811) photodetector for different case scenarios. Fig. S5. Noise for up to 2-min measurements using a 200-kHz (New Focus, 2011) photodetector. Fig. S6. Example design of [2 × 2] matrix-vector multiplication.
