# Supplementary material for: In-memory computing on a photonic platform
Source: Sci Adv. 2019 Feb 15;5(2):eaau5759. doi: 10.1126/sciadv.aau5759 (PMC6377270; doi:10.1126/sciadv.aau5759)
Supplement: http://advances.sciencemag.org/cgi/content/full/5/2/eaau5759/DC1 [file supp_5_2_eaau5759__index.html]

Science Advances | Science Advances

## Supplementary Materials

**This PDF file includes:**

- Section S1. Device characterizations using balanced splitters
- Section S2. Experimental setup
- Section S3. Noise
- Section S4. Offset correction
- Section S5. Error propagation
- Section S6. Proposed matrix-vector multiplication architecture
- Fig. S1. Balanced splitter characterization.
- Fig. S2. Diagram of the experimental pump-probe setup.
- Fig. S3. Diagram of the experimental setup for matrix-vector multiplication.
- Fig. S4. Noise for up to 20-μs measurements using a 125-MHz (New Focus, 1811) photodetector for different case scenarios.
- Fig. S5. Noise for up to 2-min measurements using a 200-kHz (New Focus, 2011) photodetector.
- Fig. S6. Example design of 2 × 2 matrix-vector multiplication.

Download PDF

**Files in this Data Supplement:**

- Adobe PDF - aau5759\_SM.pdf
