# Supplementary material for: In-memory computing on a photonic platform
Source: Sci Adv. 2019 Feb 15;5(2):eaau5759. doi: 10.1126/sciadv.aau5759 (PMC6377270; doi:10.1126/sciadv.aau5759)
Supplement: http://advances.sciencemag.org/cgi/content/full/5/2/eaau5759/DC1 [file aau5759_SM.pdf]

Science Advances
AAAS

advances.sciencemag.org/cgi/content/full/5/2/eaau5759/DC1

Supplementary Materials for

In-memory computing on a photonic platform

Carlos Ríos, Nathan Youngblood, Zengguang Cheng, Manuel Le Gallo, Wolfram H. P. Pernice, C. David Wright, Abu Sebastian*, Harish Bhaskaran*

*Corresponding author. Email: ase@zurich.ibm.com (A.S.); harish.bhaskaran@materials.ox.ac.uk (H.B.)

Published 15 February 2019, *Sci. Adv.* **5**, eaau5759 (2019)
DOI: 10.1126/sciadv.aau5759

This PDF file includes:

Section S1. Device characterizations using balanced splitters
Section S2. Experimental setup
Section S3. Noise
Section S4. Offset correction
Section S5. Error propagation
Section S6. Proposed matrix-vector multiplication architecture
Fig. S1. Balanced splitter characterization.
Fig. S2. Diagram of the experimental pump-probe setup.
Fig. S3. Diagram of the experimental setup for matrix-vector multiplication.
Fig. S4. Noise for up to 20-µs measurements using a 125-MHz (New Focus, 1811) photodetector for different case scenarios.
Fig. S5. Noise for up to 2-min measurements using a 200-kHz (New Focus, 2011) photodetector.
Fig. S6. Example design of [2 × 2] matrix-vector multiplication.

Section S1. Device characterizations using balanced splitters

Balanced-splitter devices were used to characterize the performance of the device by comparing an arm with the phase change materials with one without any. These devices offer two identical optical paths starting from a coupling grating placed in the center and employing a 50/50 beam splitter. In our experiment, the two arms of a balanced splitter with $Ge_2Sb_2Te_5$ (GST) on the right waveguide is shown in fig. S1a. To *Write*, *Erase*, *Read*, and multiply, the probe light was coupled into the right grating and out through the central port, using a wavelength of λ =1598*nm* – so this is the arm in which computations are carried out. The pump (pulses), with a wavelength of λ =1590*nm* was coupled into the device in the opposite direction. The left arm was used to characterize the attenuation by comparing the optical transmission through the arms with and without a PCM on top. The four different combinations of transmission measurements (i.e. left to center, center to right, right to center, center to left) provide information on the attenuation of the GST, the losses due to the beam splitter (i.e. if the two arms are balanced), and also allow to measure ratios between the coupling efficiency of the grating. Figure S1b shows the example of the four transmission curves for a balanced splitter device with a 1µm GST section on top. The transmission curves are limited by the bandwidth allowed by the grating couplers. In particular, each of the transmission curves can be expressed as

$$\begin{aligned} I_{1\to2} &= I_0\,\eta_1\,\beta\,\eta_2 && \text{(left to center)} \\ I_{2\to1} &= I_0\,\eta_2\,\alpha\,\eta_1 && \text{(center to left)} \\ I_{2\to3} &= I_0\,\eta_2\,\alpha\,\eta_3\,\gamma && \text{(center to right)} \\ I_{3\to2} &= I_0\,\eta_3\,\beta\,\eta_2\,\gamma && \text{(right to center)} \end{aligned} \qquad \text{(S1)}$$

where I_0 is the input signal, η_n is the efficiency of the coupling grating n, γ is the insertion loss of the GST cell, α is the insertion loss of the beam splitter when coupling from the central grating (grating 2), and β is the insertion loss of the beam splitter when coupling from the right or left grating (gratings 1 and 3). From Equations S1, the different parameters that characterize the device can be found

$$\begin{aligned} I_{3\to2}/I_{1\to2} &= \eta_3\gamma\,/\,\eta_1 \\ I_{2\to3}/I_{2\to1} &= \eta_3\gamma\,/\,\eta_1 \\ \\ I_{3\to2}/I_{2\to3} &= \beta\,/\alpha \\ I_{1\to2}/I_{2\to1} &= \beta\,/\alpha \end{aligned} \qquad \text{(S2)}$$

Figure S1c shows the results of the calculations shown in Eq. S2 using the data in fig. S1a for balanced splitter with a $1\mu m$ long GST. In particular we found in this device that $\beta = \alpha$ for all wavelengths and from two different measurements (blue and green lines), which means that both arms are well balanced. The BS splits 50/50 when coupling from the central port but also loses half of the energy when coupling from left or right and measuring in the central grating. We also measured the insertion loss of the GST memory cell, which varies with the wavelength. Good agreement was found between the measurements from the two arms (black and red curves) which implies that the grating couplers have similar efficiencies, which were also calculated from fig. S1b by knowing the input probe power. All this information was used to calculate the pulse energies before and after the PCM as well as the switching energies in different devices, some of which were not balanced.

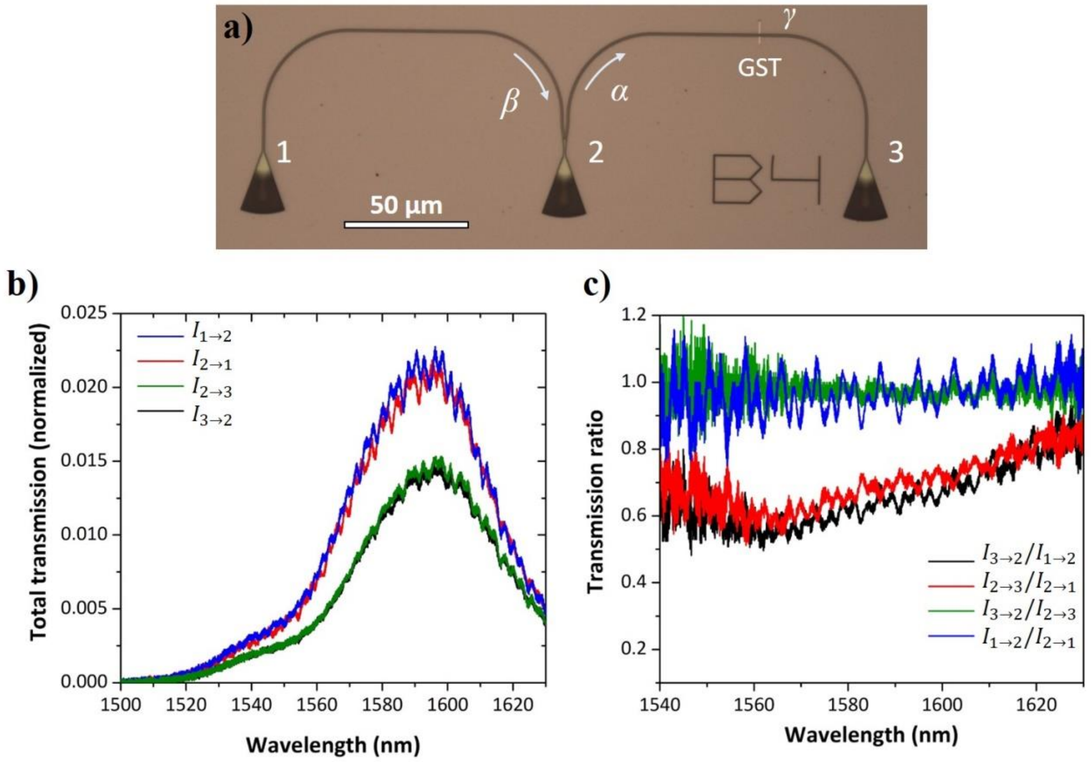

Fig. S1. Balanced splitter characterization. a) optical microscope image of a balanced splitter with a 5μm long GST section covering the entire waveguide. The numbers correspond to each of the gratings. γ is the insertion loss of the GST cell, α is the insertion loss of the beam splitter when coupling from the central grating (grating 2), and β is the insertion loss of the beam splitter when coupling from the right or left grating (gratings 1 and 3 toward 2). **b**) Normalized transmission curves as a function of the wavelength for the four possible combinations of the three ports in a balanced splitter device. **c**) Calculation of the device parameters from Eq. S2, from these results, $\alpha, \beta, \gamma,$ and the relation between two of the different η (grating efficiency – more data is required to calculate them exactly) can be calculated.

Section S2. Experimental setup

Figure S2 shows the optical pump-probe setup employed in all the measurements of this work, which is described in the Methods and Materials Section of the main paper. Pump (green line) and probe (blue line) are generated from two different CW lasers in the telecommunications band. The pump is pulsed by using an electro-optical modulator (EOM) and then amplified with an erbium-doped fiber amplifier (EDFA). To reduce the noise coming from spontaneous emissions at the EDFA, a wavelength filter is added to the pump channel. A beam splitter is used to get a reference of the input from the 10% of the pump pulse using a 125MHz detector. The remaining 90% is coupled into the chip, whose transmission, past the PCM memory cell, is measured employing a 1 GHz photodetector. The probe, on the other hand, is polarized to match the TEM mode of the gratings and integrated waveguides, and then coupled into the chip. The output signal of the probe is split into two

beams to measure both the real-time dynamics with a 125MHz photodetector and the long-term transmission with a 200KHz low-noise photoreceiver.

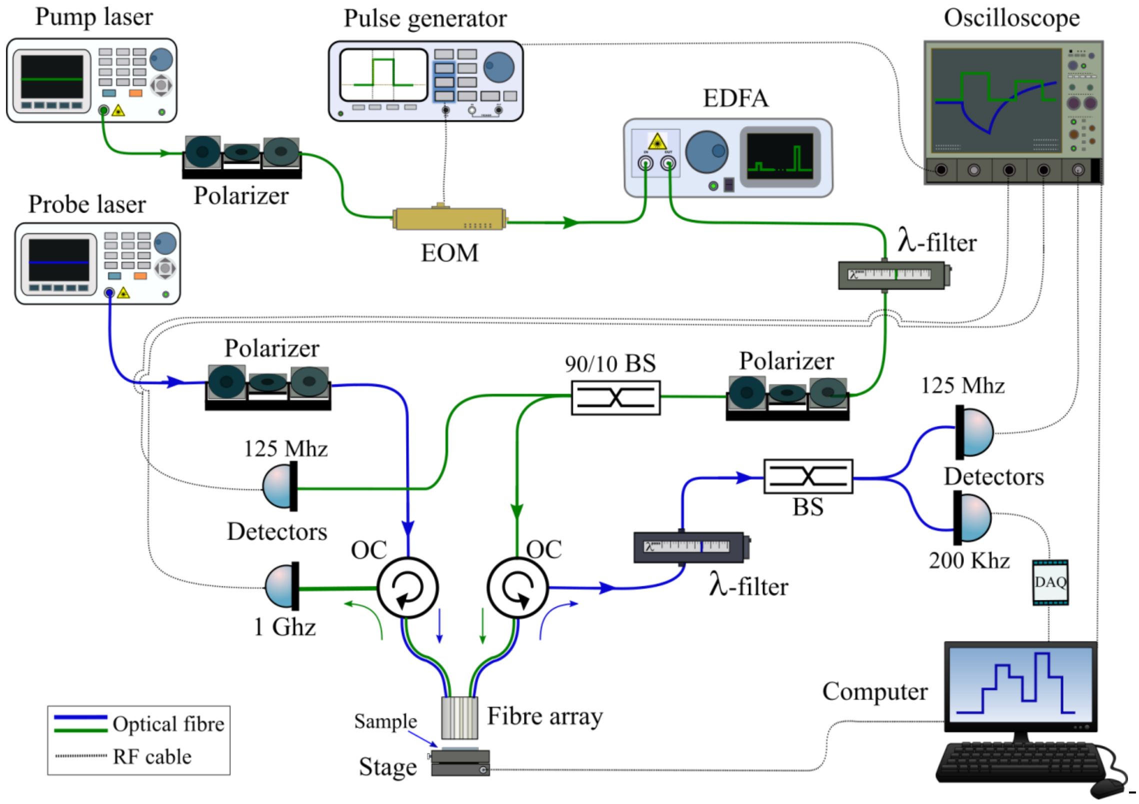

Fig. S2. Diagram of the experimental pump-probe setup. Here, EOM stands for electro-optical modulator, BS for Beam splitter, OC for Optical circulator, EDFA for erbium-doped fibre amplifier and DAQ for data acquisition unit. λ-filter represents a wavelength-tunable filter.

We upgraded the setup to include a third laser to obtain a second pump pulse as shown in fig. S3. This system is used to control the crystallographic state of two GST memory cells simultaneously. In this case, we remove the real-time analysis and use the CW probe only to record the transmission levels of both GST measured at the output of the left and right grating couplers with 200 khz detectors. Moreover, we obtain the information of the input and output pulses by measuring before the EDFA and at the output of the central grating coupler, respectively. The output pulse from the central grating corresponds to the sum of the two scalar multiplications as shown in Fig. 5d in the main text.

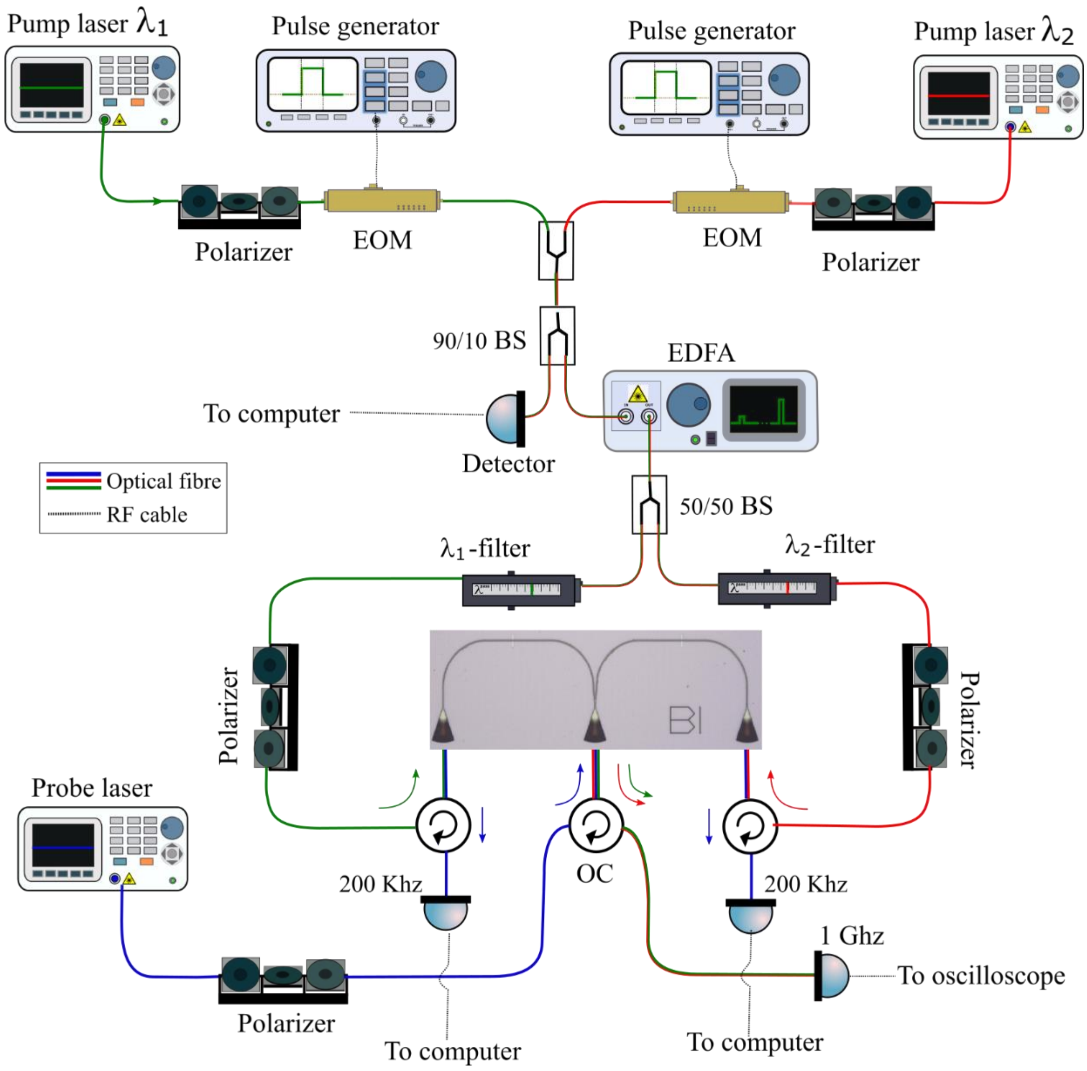

Fig. S3. Diagram of the experimental setup for matrix-vector multiplication. Here, EOM stands for electro-optical modulator, BS for Beam splitter, OC for Optical circulator, EDFA for erbium-doped fibre amplifier, and λ-filter represents a wavelength-tunable filter.

Section S3. Noise

We measured the optical transmission through integrated photonic waveguides, with and without GST on top, to study the sources of noise from the Fourier transform of the signals. This experiment was realized in two different timescales, seconds and microseconds, with a 200 kHz (New Focus, 2011) and a 125MHz (New Focus, 1811) photodetector, respectively. A control measurement without power was also carried out to obtain the effect of the dark current. Figure S4 shows the results of measuring during 20µs with the 125MHz detector for different scenarios. We recorded the data at the oscilloscope for single shot and ten signals average measurements. Other than the differences in amplitude due to the SNR changes as the probe power increases, we observed no difference in the spectra. In particular, we did not find a significant difference between the spectra of measurements with GST and those with the photodetector in OFF. This implies that the transmission level is stable over the time the measurements was carried out. Moreover, the noise is ultimately given by the electronics of

the photodetector. In the experiment, the noise can be reduced by increasing the SNR using a higher probe values and averaging signals.

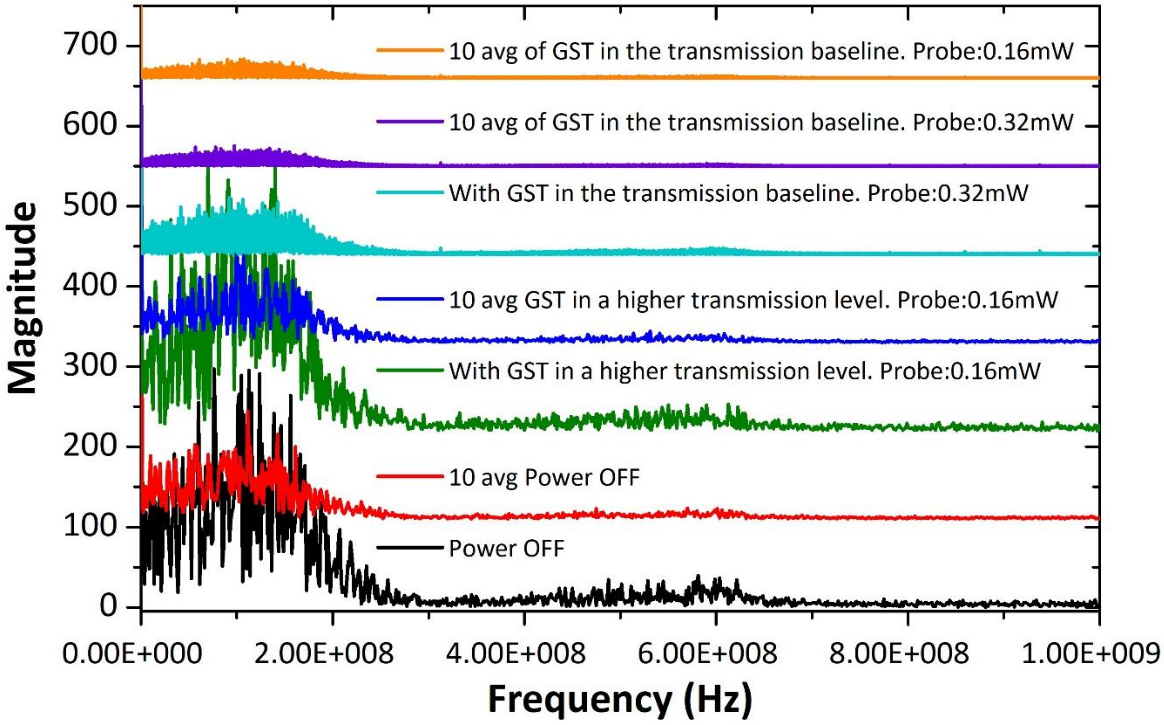

Fig. S4. Noise for up to 20-μs measurements using a 125-MHz (New Focus, 1811) photodetector for different case scenarios. The probe power corresponds to the power inside the waveguide, before the GST. The high transmission level refers to the maximum transmission level achieved by amorphizing GST (measurement was carried out after the transition was triggered).

We repeated the experiment in the seconds timescale using a 200kHz detector. We measured during 2min the transmission signal at the output of our devices for different cases: GST in the baseline and in a high-transmission levels, power OFF at photodetector for dark current, and for a device with no GST. The results, shown in fig. S5, lead to similar conclusions. The noise is governed by the dark current at the detector with very well defined peaks that repeat over all the measurements. Not having variations in the spectrum means that, at least for the baseline, there is no optical drift and the level stability is guaranteed over time. The only different situation in this case took place for the measurement of a device with GST in a high transmission level (which included also the instant of the switching, which is not properly resolved at 200kHz rate). In this case, low frequencies present a large magnitude as result of a slow change in the transmission level. We found that this effect is not due to the relaxation of the GST, which as demonstrated in the main paper in Fig. 3 does not take place, but rather to the fact that a probe of 0.16mW induces recrystallization at a very slow rate (out of range for the measurements in fig. S3) as this power is enough to slowly increase the temperature, above 150°C, of small portions of GST. This situation was corrected by reducing the probe

power to 0.05mW, as described in the main text, in which case no drift was observed and therefore, no low frequencies in the Fourier transform.

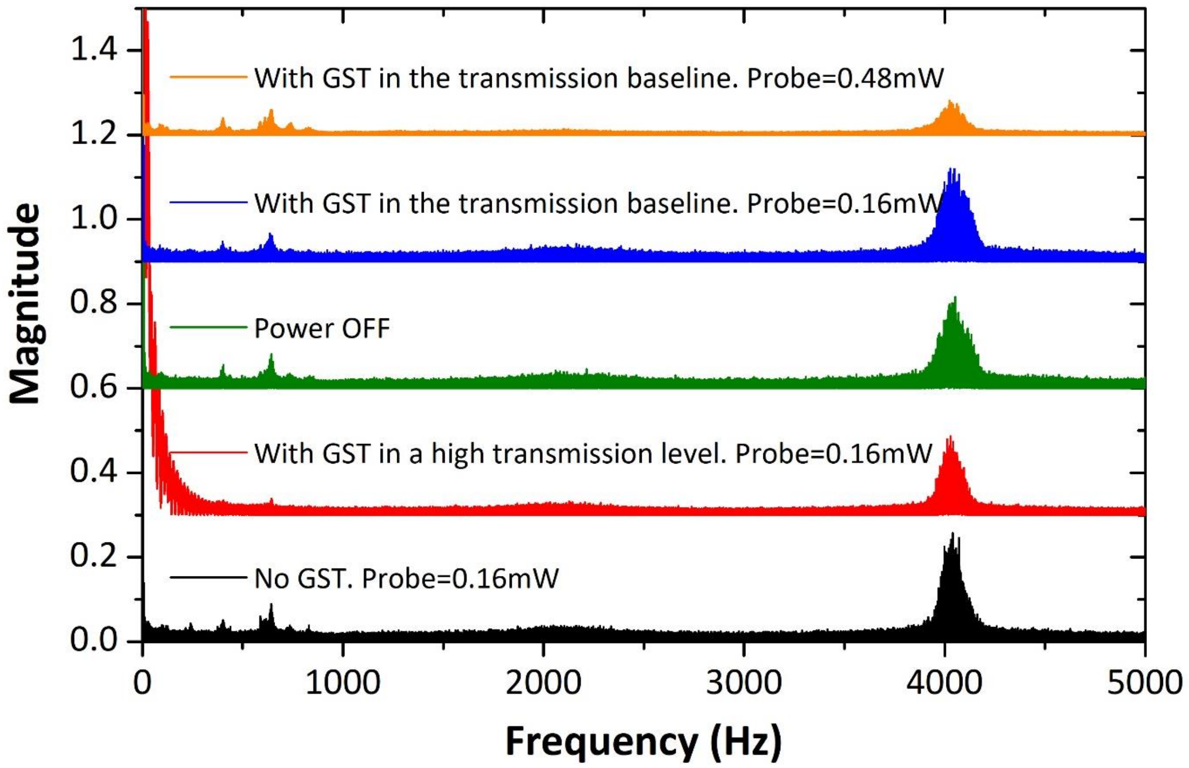

Fig. S5. Noise for up to 2-min measurements using a 200-kHz (New Focus, 2011) photodetector. The probe power corresponds to the power inside the waveguide, before the GST. The high transmission level refers to the maximum transmission level achieved by amorphizing GST (measurement was carried before, during, and after the transition was triggered).

Section S4. Offset correction

When an input pulse propagates through a PCM memory cell, the output power is equal to $P_{out} = T(P_{\mathrm{Write}}) \times P_{in}$, where T corresponds to the transmittance level define by a *Write* pulse P_{Write} in a previous step. However, T can be expressed as $T(P_{\mathrm{Write}}) = \Delta T + T_0$, where $\Delta T = T - T_0$ corresponds to the change in transmission as function of the P_{Write} pulse and T_0 is the baseline transmission level (fully crystalline). Therefore, the real output pulse is

$$P_{out} = \Delta T \times P_{\mathrm{in}} + T_0 \times P_{in}$$

With ΔT linearly mapped to a number $a \in [0, 1]$ and P_{in} to $b \in [0, 1]$, $P_{out} = a \times b \in [0, 1]$ only if the offset term $T_0 \times P_{in}$ is removed. This offset can be easily calculated by characterizing T_0 for each memory cell, then a post processing is required to remove it from

the experimental value. With the offset correction, we have that the final measured value $P'_{out} = \Delta T \times P_{\mathrm{in}}$ and the *measured* result of the multiplication corresponds to

$$a \times b = \frac{\Delta T \times P_{\mathrm{in}}}{\max(\Delta T) \times \max(P_{\mathrm{in}})} = \frac{P'_{out}}{\max(P'_{out})} \in [0, 1]$$

This last relation holds as long as the multiplication 1×1 is carried out to obtain the right normalization (i.e. if $\max(P_{out}) = \max(\Delta T) \times \max(P_{\mathrm{in}})$ is measured for each device). This way the measured value can be obtained without further post processing. Moreover, to calculate the *exact*, ΔT can be also normalized with respect to T_0, i.e. $\Delta T' = \Delta T / T_0 = (T - T_0)/T_0$, such that the changes in transmission in percentage can be used (as plotted in Fig. 2c in the main text). Given than in the normalization to the $[0, 1]$ range the division by the constant T_0 takes place in both the numerator and denominator, the result is not altered.

Section S5. Error propagation

As plotted in Fig. 2e in the main text, the error in the programmed level was given by a standard deviation of 0.35% (with respect the total transmission change) by using a normal distribution over all the data collected. This error is due to the fluctuations of the input pulse powers as result of electronic latencies in the EOM—the pulse power was tuned by changing the voltage on the EOM which we found induces fluctuations, the same voltage may not result in exactly the same pulse. This adds uncertainty in reaching a transmission level and sending the right P_{in} to the PCM memory cell, which in turns result in errors for the scalar multiplication. Considering errors ε_1 and ε_2 associated to the standard deviation of the pulse power after normalization to the {0,1} range, the multiplication $a \times b$ is then given by

$$a \times b \to a(1 \pm \varepsilon_1) \times b(1 \pm \varepsilon_2) = ab \pm ab(\varepsilon_1 \pm \varepsilon_2 \pm \cancel{\varepsilon_1\varepsilon_2})$$

this means that the error in the multiplication will increase linearly (assuming $\varepsilon_1\varepsilon_2 \approx 0$) as a function of the multiplication of the numbers a and b, which can be observed in the broadening of the Fig.4c for values close to 1 and quantitatively in the inset of Fig. 4d. This broadening can be reduced by having more reliable sources of identical pulses.

Section S6. Proposed matrix-vector multiplication architecture

The matrix-vector multiplication shown in fig. S6 can be generalized to multiplication between a [$k \times N$] matrix and [$N \times 1$] vector using multiple cascaded power splitters. Figure S5 shows the exemplary structure for a multiplication between a [2×2] matrix and a [2×1] vector.

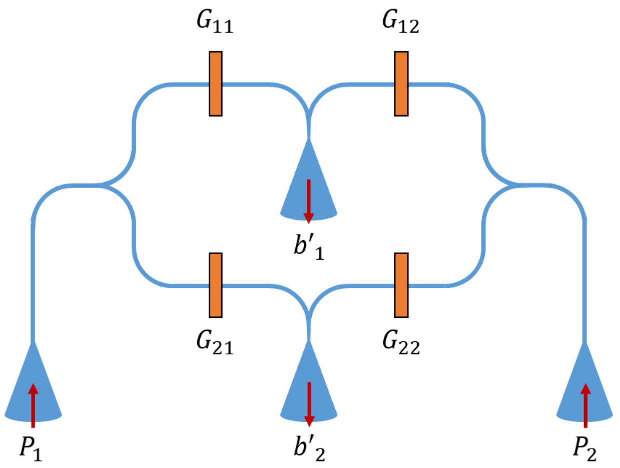

$$\frac{1}{4}\begin{bmatrix} G_{11} & G_{12} \\ G_{21} & G_{22} \end{bmatrix}\begin{bmatrix} P_1 \\ P_2 \end{bmatrix} = \begin{bmatrix} b'_1 \\ b'_2 \end{bmatrix}$$

Fig. S6. Example design of [2 × 2] matrix-vector multiplication. An example photonic circuit that implements multiplication between a [2 × 2] matrix and a [2 × 1] vector.
